# Policy implications of marked reversals of population life expectancy caused by substance use

**DOI:** 10.1186/s12916-016-0590-x

**Published:** 2016-03-10

**Authors:** Jürgen Rehm, Peter Anderson, Benedikt Fischer, Antoni Gual, Robin Room

**Affiliations:** Social and Epidemiological Research (SER) Department, Centre for Addiction and Mental Health (CAMH), 33 Russell Street, T505, Toronto, ON M5S 2S1 Canada; Campbell Family Mental Health Research Institute, CAMH, 250 College Street, Toronto, ON M5T 1R8 Canada; Dalla Lana School of Public Health, University of Toronto, 155 College Street, 6th floor, Toronto, ON M5T 3M7 Canada; Department of Psychiatry, University of Toronto, 250 College Street, Toronto, ON M5T 1R8 Canada; Institute of Medical Science, University of Toronto, 1 King’s College Circle, Toronto, ON M5S 1A8 Canada; PAHO/WHO Collaborating Centre, CAMH, 33 Russell Street, Toronto, ON M5S 2S1 Canada; Epidemiological Research Unit, Klinische Psychologie & Psychotherapie, Technische Universität Dresden, Chemnitzer Str. 46, 01187 Dresden, Germany; Institute of Health and Society, Newcastle University, Newcastle, NE2 4AX UK; School for Public Health and Primary Care, Maastricht University, Maastricht, 6200 MD The Netherlands; Centre for Criminology and Socio-legal Studies, University of Toronto, 14 Queen’s Park Cres. W., Toronto, ON M5S 3K9 Canada; Grup de Recerca en Addiccions Clínic, Institut Clínic de Neurosciències, Villarroel, 170, 08036 Barcelona, Spain; Institut d’Investigacions Biomèdiques Agustí Pi i Sunyer, IDIBAPS, Rosselló, 149-153, 08036 Barcelona, Spain; Centre for Alcohol Policy Research, La Trobe University, 215 Franklin St., Melbourne, Victoria 3000 Australia; Centre for Social Research on Alcohol and Drugs, Stockholm University, Sveaplan, 106 91 Stockholm, Sweden; Melbourne School of Population and Global Health, University of Melbourne, Carlton, 207 Bouverie St., Victoria, 3010 Australia

**Keywords:** Life expectancy, Marked reversals of trend, Substance use, Policy, Public health

## Abstract

**Background:**

Life expectancy has been increasing steadily over the past century in most countries, with only a few exceptions such as during wartimes.

**Discussion:**

Marked reversal of life expectancy has been linked to substance use and related policies. Three such examples are discussed herein, namely the double reversal of life expectancy trends (first to positive, then to negative) associated with reducing alcohol supply in the then Union of Soviet Socialist Republics (USSR), followed by a rapid increase in availability; the impact of the rapid increase of prescription opioids on white non-Hispanics in the US; and the systemic impact of the violence accompanying the drug war in Mexico on the life expectancy of men. Alcohol policies were crucial to initiate the positive reversal in the USSR, and different substance use policies could have avoided the negative impacts on life expectancy of the described large groups or nations.

**Summary:**

Substance use policies can be responsible for abrupt negative changes in life expectancies. An orientation of such policies towards the goals of public health and societal well-being can help avoid such changes.

## Background

Life expectancy has increased steadily over the past century, except during mass pandemics, such as the influenza pandemic of 1918/1919, or during World Wars I and II [1–3] (see also: http://vizhub.healthdata.org/le/ accessed March 3, 2016). For most countries, the upward trend has remained uninterrupted since the end of World War II. These gradual upward transitions of life expectancy are based on an environment composed of a complex interplay of a variety of major risk and protective factors [4], with the balance improving steadily in the overwhelming majority of countries [1–3]. Tobacco, alcohol, and illicit drug use are part of these environments; all three categories of substances are among the top 20 risk factors globally, with most of the burden of disease and mortality attributable to tobacco followed by alcohol, with drugs as a distant third [4]. Thus, substance use has been known to have a negative impact on life expectancy and burden of disease, but usually these impacts are counterbalanced by more positive impact factors.

Abrupt changes of directions in life expectancy have been rare, usually triggered by reversal of mortality rates in mid-adulthood, and linked to specific events, such as the aforementioned pandemic or wartimes [3]. However, substance use policies can also create sudden or relatively abrupt population impacts, as will be illustrated herein with three examples.

## Examples of marked reversals of life expectancy linked to substance use policies

One, almost classic, example for abrupt reversals in life expectancy relates to the changes associated with the alcohol reforms of the Union of Soviet Socialist Republics in the Gorbachev era, which reversed a negative trend in life expectancy into a positive one following a reduction in the supply of alcohol (increase in life expectancy from 1984 to 1987: men, 3.2 years; women, 1.3 years [5, 6]). When the restrictive alcohol policy was abandoned and alcohol became widely available, the trend in life expectancies reverted once again (decrease in life expectancy between 1987 and 1994: men, 7.3 years; women, 3.3 years [5, 7]). Obviously, these figures represent associations between availability and consumption of alcohol with life expectancy rather than a proof of causality, however, at least for the first reversal, it would be hard to find other explanations, as the increases in life expectancy occurred during a time of economic crisis with no plausible alternative explanations. For the negative trend reversal, attribution of causality is less clear, as this change took place in a period of massive privatization (e.g. [8]) and economic decline, with resulting unemployment (e.g. [9, 10]), community destabilization, subsequent psychological stress (e.g. [11]), and increasing inequality [12]. Some of these variables were included in the most comprehensive econometric modeling on the Russian experience of rapidly decreasing life expectancy [7]. Moreover, alcohol-attributable causes of death were especially impacted in both reversals for life expectancy [5, 7]. Finally, more recent examples of the effects of alcohol policies on mortality confirmed the importance of alcohol policies in the Russian case [13, 14]. In terms of mechanisms, the underlying policy interventions to reduce mortality were mainly alcohol availability restrictions and taxation [5, 13], the latter only for more recent interventions.

The second example concerns drug policy, and specifically policies for regulating prescription opioids (POs) in the United States (US). Overall, larger quantities of POs are used in the US than in any other country (in the latest available statistic for 2011–2013, Canada was a distant second, with about 60 % of the consumption per capita of the US [15]). The rapid increase of PO use and misuse commenced in the mid-1990s, in part by allowing family doctors to prescribe short-acting opioids like oxycodone for relatively common disease categories such as chronic pain [16, 17]. With increased availability of POs, non-medical use increased proportionally along with their associated harm, such as overdose deaths (albeit with some lag) [16, 18–20], contributing to a reversal of all-cause mortality of middle-aged white non-Hispanics in the US [21]. While the mortality trend for the general population was steadily decreasing prior to 1999, between 1999 and 2013 mortality increased by 9 % in white non-Hispanics, but continued to decrease in black non-Hispanics and Hispanics. Again, this is an association, but the increase in mortality for white non-Hispanic middle aged Americans was largely accounted for by increasing death rates from drug and alcohol poisoning deaths, suicide, and chronic liver diseases and cirrhosis, all directly or indirectly associated with substance use and substance use policies (drug and alcohol poisoning deaths per definition; suicide [22]; liver cirrhosis [23]; causal links for illicit drugs in general [24]). POs played the major role in this mortality mix, as prescription overdose deaths have become the most prevalent form of overdose death in the past decade in North America, accounting for approximately 40 % of the total drug poisoning deaths [25–27]. The rise in heroin overdose deaths in recent years can also be partly attributed to use initiated by way of previous PO use [28].

The third example, also arising from illicit drug policies, relates to the systemic consequences of substance use policies (for a definition see [29]). A recent paper showed that, after six decades of gains in life expectancy for Mexico, the trend stagnated for the period following the year 2000 and, for men after 2005, it actually reversed [30]. This reversal of trends in life expectancy was mainly caused by an unprecedented rise in homicide rates, in large part linked to illicit drugs and the war on drugs, i.e. linked to gang wars and/or conflicts between drug gangs and police or the army [31]. In general, enforcement of prohibitive drug laws has been shown to impact adversely on drug market violence in a systematic review of the evidence [32], and alternative regulatory models will be required if drug supply and drug market violence are to be meaningfully reduced [33].

## Conclusions

These examples are chosen to demonstrate that changes in substance use, unlike changes in other risk factors, can affect population life expectancy not only in the long term but also abruptly, reversing decade-long trends. As shown, this is even true for illicit drugs, which have been linked to much less overall mortality and burden of disease than legal substances such as alcohol and tobacco (see above and [4]). The cases cited represent dramatic changes in policies and use patterns, where the connections with overall disease burden are striking. However, there is also ample evidence that appropriate incremental changes in policy or their enforcement have had effects on health outcomes [34, 35].

The health impact in the dramatic cases cited above show that substance use policy decisions can have substantial effects on the burden of disease if policymakers get it wrong; however, they also point to potential substantial benefits if policymakers get it right, i.e. if they establish policies associated with a positive impact on population well-being and the burden of disease, including mortality [33]. Several principles have been identified to allow for such a positive transformation:

- There should be active monitoring of substance-attributable disease burden and mortality. Identifying rapid changes in substance-attributable causes of death above a certain size will prepare the way for adequate policy changes (see [36], as example for alcohol).
- Active and integrated substance use policies should be created, oriented at public health gains as a major goal, and with decriminalization of substance use [33, 37] (see also the UNAIDS recommendations for the United Nations General Assembly Special Session on the World Drug Problem [38]). Substance use policies must include legal and illegal substances and psychoactive medications, as evidenced by the second example [35]. The public health approach explicitly includes considerations about harm to others attributable to substance use (i.e. second-hand smoke, effects of substance use on road traffic and operating machinery, violence, effects on the family).
- Regulation of availability of substances, including regulation of affordability, is one of the cornerstones for substance use policies (see above and [34, 35, 39]).
- Trade agreements and dispute mechanisms – global, regional, and bilateral – need to be changed so that market restrictions on legally traded psychoactive substances for public health purposes cannot be challenged or nullified [40, 41].
- Access to treatment and social assistance for heavy users and their families should be improved, which needs to be linked to a reduction of stigmatization. Substance use disorders are the least treated mental conditions, and mental conditions as a whole are less treated than somatic conditions [42]. Improving access to treatment and social assistance would also help in achieving the UN sustainable development goal, specific target 3.5, asking for a strengthening of “*prevention and treatment of substance abuse*” [43].
- Policy responses should be relative to the potential of substances to reduce well-being, including, but not limited to, burden of disease and mortality [33, 44].
